# Achieving global targets on breastfeeding in Thailand: gap analysis and solutions

**DOI:** 10.1186/s13006-021-00386-0

**Published:** 2021-05-07

**Authors:** Chompoonut Topothai, Viroj Tangcharoensathien

**Affiliations:** 1 International Health Policy Program, Ministry of Public Health, Nonthaburi, Thailand; 2 Bureau of Health Promotion, Department of Health, Ministry of Public Health, Nonthaburi, Thailand

**Keywords:** Breastfeeding, Policy, Thailand, Infant and young child feeding

## Abstract

**Background:**

Global advocates for breastfeeding were evident since the International Code of Marketing of Breast-Milk Substitutes (BMS Code) was adopted in 1981 and fostered by subsequent relevant World Health Assembly resolutions, using a framework that promotes, supports and protects breastfeeding. Global partners provided comprehensive support for countries to achieve breastfeeding targets while progress was closely monitored. This review identifies breastfeeding policy and implementation gaps in Thailand.

**Main findings:**

Although Thailand implemented three Thai voluntary BMS Codes, ineffective enforcement results in constant violations by BMS industries. In light of strong resistance by the BMS industries and their proxies, it was not until 2017 that the Code was legislated into national law; however regulatory enforcement is a protracted challenge. A Baby-Friendly Hospital Initiative (BFHI), mostly in public hospitals, was successfully applied and scaled up nationwide in 1992, but it later became inactive due to lack of continued support. Several community-based and workplace programmes, which supported breastfeeding, also faced challenges from competing agendas. Although the Labor Protection Law offers 98 days maternity leave with full pay, the conducive environment for successful six- month exclusive breastfeeding (EBF) needs a significant boost. These gaps in policy were exacerbated by a lack of multi-sectoral collaboration, ineffective implementation of existing interventions, inadequate investment, and lack of political will to legislate six-month maternity leave.

As a result, the progress of EBF rate during the first 6 months as measured by previous 24 h was erratic; it increased from 12.3% in 2012 to 23.1% in 2015 and decreased to 14% in 2019. There was a deterioration of early initiation from 49.6% in 2006 to 34% in 2019. These low performances hamper the achievement of global targets by 2030.

**Conclusions:**

We recommend the following. First, increase financial and human resource investment, and support successful exclusive breastfeeding in BHFI, communities and workplaces through multi-sectoral actions for health. Second, implement the active surveillance of violations and strengthen law enforcement for timely legal sanctions of violators. Third, revitalize the BFHI implementation in public hospitals and extend to private hospitals.

## Background

Evidence shows that breastfeeding generates high returns on various dimensions such as health, economic, social, and the environment [1]. It is estimated that every US dollar invested in improving breastfeeding practice could result in a $35 US dollar return [2]. Current inadequate breastfeeding rates cause an estimated $302 billion US dollar annual economic loss from preventable illnesses and healthcare costs for treatment; this is equivalent to 0.49% of the World’s Gross National Income [3].

The high return on investment of breastfeeding is mainly from the positive health impact in children and mothers. Breastfeeding protects children from infection-related mortality, reduces the odds of non-communicable disease (NCD) particularly overweight and obesity, and stimulates cognitive development [4]. In mothers, a longer period of breastfeeding throughout a lifetime is associated with a reduction in the odds of developing breast and ovarian cancers [1]. In terms of environment, breastfeeding does not generate greenhouse gases or a carbon footprint and waste, compared with infant formula feeding [5].

Despite the obvious benefits of breastfeeding, globally only 43% of infants were breastfed within 1 h after birth, 41% exclusively breastfed during the first 6 months, and 45% breastfed at 2 years of age [3]. The current rate is still low and too distant from the global target set by World Health Organization (WHO), United Nation Children’s Fund (UNICEF), and global partners; this is at least 70% of children breastfed within 1 h of birth, 70% of children exclusively breastfed during the first 6 months (as measured by 24 h recall period), and at least 60% of children continuing to breastfeed at 2 years of age by 2030 [3].

Breastfeeding rates in Thailand are even lower than the global average and seem to be off track to achieve the global target, even though various interventions have been implemented [6]. This study reviewed the work of global breastfeeding advocates, assessed progress and identified gaps in Thailand’s breastfeeding policy and implementation in three dimensions: promote, support and protect. Relevant publications and documents retrieved from worldwide databases and Google scholar were reviewed. Lessons learned from Thailand can inform other low- and middle-income countries to support meeting breastfeeding targets by 2030.

### Global advocacy and recommendations on breastfeeding: a historical milestone

The advancement of global advocacy and recommendations on breastfeeding is rooted in the adoption of the International Code of Marketing of Breast-Milk Substitutes (BMS Code) at the 34th World Health Assembly (WHA) in 1981 [7, 8]. During 1981–2000, the concept of ‘protection, promotion, and support of breastfeeding’ serves as a framework for countries to implement necessary policy and programmes. Twenty years later, however, the focus is on setting global targets, designing effective strategies, building networks, and developing action plans for accelerating and monitoring progress.

The BMS Code and subsequent WHA resolutions brought global attention to the marketing practices of breast milk substitute (BMS) companies, aiming to ensure that information given to mothers is appropriate for making informed decisions about when to use BMS products [9]. Eight years after the adoption of the BMS code, breastfeeding was acknowledged as a child’s right in the Convention on the Right of the Child (CRC) adopted by United Nations General Assembly in 1989 [10] in Article 24, 2 (e): “To ensure that all segments of society, in particular parents and children, are informed, have access to education and are supported in the use of basic knowledge of child health and nutrition, the advantages of breastfeeding, hygiene and environmental sanitation and the prevention of accidents”. The CRC has sent strong message which calls for collective societal responsibilities towards breastfeeding [11].

Subsequently, the Innocenti Declaration on the Protection, Promotion and Support of Breastfeeding was produced and declared in 1990 as an agreement adopted by the participants at the WHO/UNICEF policymakers’ meeting on “Breastfeeding in the 1990s: A Global Initiative” held at the Ospedale degli Innocenti, Florence, Italy [12]. This innovation created policy momentum and commitment at country level to develop national breastfeeding policies and set appropriate national targets for the 1990s, as well as articulate the roles of international organizations in monitoring and providing technical support for country programmes. Following the Innocenti Declaration of 1990, the Baby-Friendly Hospital Initiative (BFHI) was launched by WHO and UNICEF in 1991 with a recommendation of Ten Steps to Successful Breastfeeding [13]. BFHI provided essential tools, materials, and steps for healthcare facilities to promote awareness of breastfeeding and the support and protection of babies, mothers and families. The BFHI was an entry point for integrating breastfeeding awareness into maternity service systems [14].

Later on, the Maternity Protection Convention number 183 was adopted by the General Conference of the International Labour Organization (ILO) in 2000 in order to further promote equality of women in workforce as well as health protection for mothers and children through the adoption of national legislations and regulations [15].

In 2002, the Global Strategy for Infant and Young Child Feeding was endorsed by WHA resolution 55.25; this milestone urged countries to adopt six-month exclusive breastfeeding which is a cost-effective intervention [16]. In 2012, a comprehensive implementation plan on maternal, infant, and young child nutrition was endorsed by the WHA resolution 65.6 [17]. This plan reiterated the importance of breastfeeding by introducing a breastfeeding target as one of six global targets on child nutrition; at least 50% of children should be exclusively breastfed during the first 6 months [18].

**Fig. 1:**
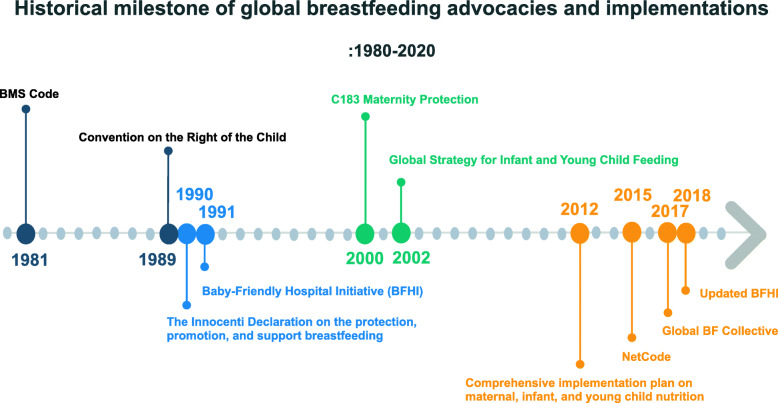
Historical milestone of global breastfeeding advocacies and implementations, four decades between 1980 and 2020

Networking and political advocacy have been a major focus during the last 5 years, 2015–2020. In 2015, the Network for Global Monitoring and Support for Implementing BMS Code namely ‘NetCode’ began to play a significant role in monitoring progress and supporting the enactment of BMS Code into national law [19]. NetCode was followed by the launch of the Global Breastfeeding Collective in 2017, that provided technical, financial, emotional and public support of breastfeeding by promoting the seven most recommended tools [20, 21]. Most recently in 2018, WHO and UNICEF launched revised guidance for the implementation of Baby-Friendly Hospital Initiative [22]. Figure 1 summarizes the milestone of global breastfeeding advocacies and implementations in the last four decades.

**Fig. 2:**
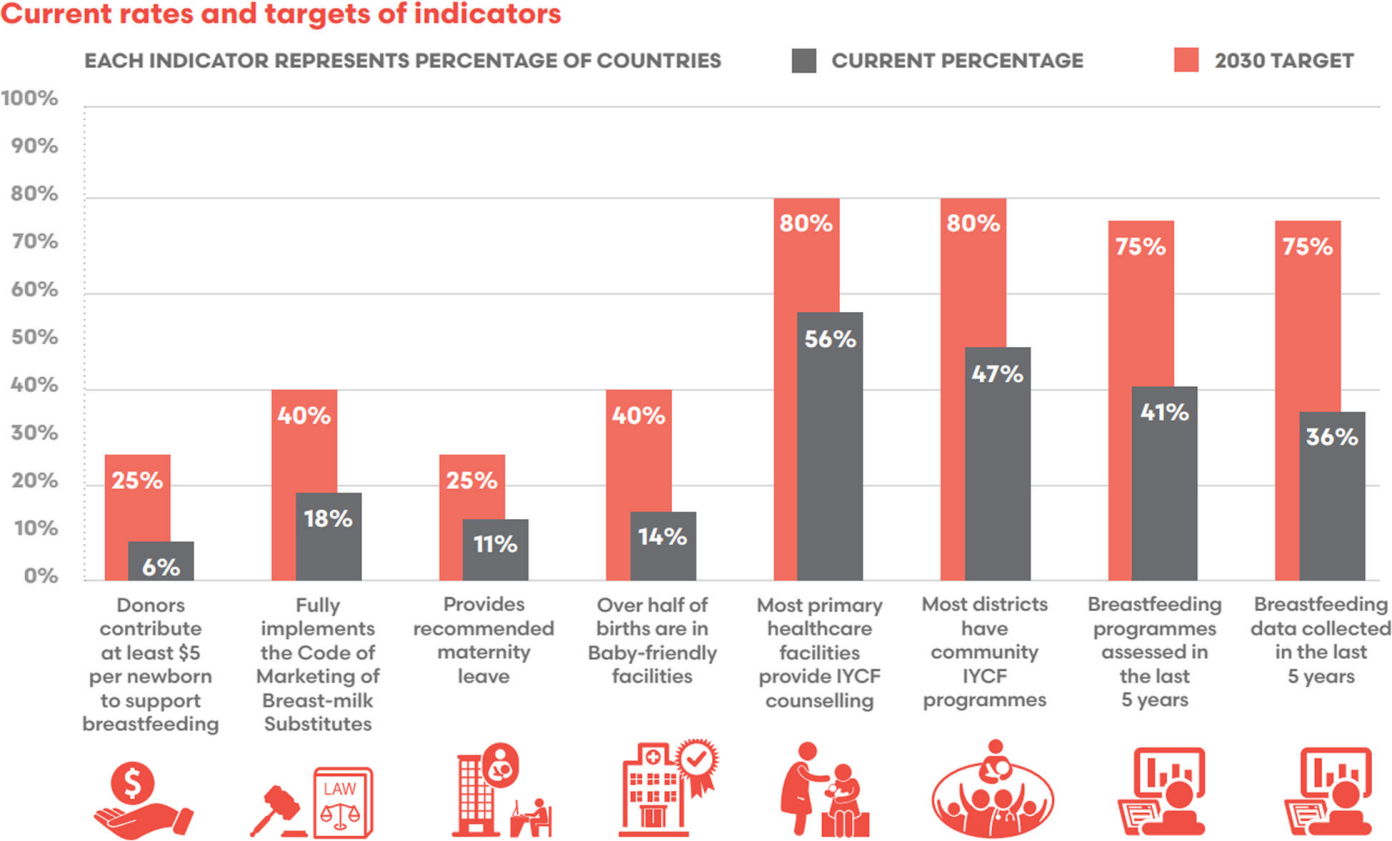
Current achievement against targets of global breastfeeding collective’ indicators. Source: Global Breastfeeding scorecard 2019, page 2 [3]

However, the 2019 Global Breastfeeding Collective report showed that a small number of countries had progressed in advocating and implementing recommended breastfeeding policy and programmes [3]. Figure 2 presents the achievement against the 2030 targets based on global breastfeeding collective’ tools.

### Thailand’s commitment to breastfeeding: promote, support and protect

The momentum of global breastfeeding advocacy has reached Thailand. Immediately after the 1990 Innocenti Declaration and the 1991 launch of BFHI, Thailand’s national breastfeeding project was established in 1992. Its objective was to empower all women to breastfeed their children exclusively for the first four to 6 months and continued breastfeeding with complementary food up to the age of 2 years or beyond. The national target of 15% of infants being exclusively breastfed for at least 4 months was also set to be achieved by 2001 [23].

#### Breastfeeding promotion

The initial focus in 1992 was breastfeeding promotion through the establishment of BFHI. All public hospitals were encouraged to deliver services according to the “Ten Steps to Successful Breastfeeding”. Various training activities and relevant information were provided to all hospitals to ensure their awareness and compliance. The BFHI was scaled up nationwide in 1995 with support from WHO and UNICEF [23]. The Ministry of Public health (MOPH) strengthened BFHI and almost all public hospitals were accredited as BFHI hospitals in 1997 [24]. After 3 years of BFHI implementation, evaluation showed improvement in breastfeeding coverage as 4-month predominant breastfeeding increased to 30% in 1998 from 19% in 1993 [23]. In 2003, the MOPH announced a national policy for six-months exclusive breastfeeding (instead of the previous target of four to 6 months) and continued breastfeeding up to 2 years of age or beyond, in line with recommendations in the Global Strategy of Infant and Young Child Feeding [23].

Subsequently, a newly national project under the royal family patronage - namely ‘Family Love Bonding Project’ (FLBP) - was launched in 2005 with the aim to promote breastfeeding practice and child development. The Baby-Friendly Hospital Initiative was integrated as a fundamental principle of Family Love Bonding Project [24]. Lactation management system and training of nurses and health professionals were initiated and strengthened nationwide. Additional resources were allocated to support the implementation of FLBP. Assessment and accreditation were conducted regularly. Finally, the FLBP ended in 2015 when all public hospitals adopted BFHI’s Ten Steps for Successful Breastfeeding into their routine practices.

#### Breastfeeding support

In 2006, the sub-district breastfeeding support programme was initiated with full engagement by communities and village health volunteers through home visits and basic care for all lactating mothers [24]. In parallel, ‘the breastfeeding corner in the workplace project’ has been initiated by the Department of Labour Protection and Welfare, Ministry of Labour, in collaboration with the Department of Health, Ministry of Public Health, and the Thai Breastfeeding Center Foundation (TBCF). This project aimed not only to support breastfeeding for lactating mothers after returning to work using a breastfeeding corner, but also to raise employers’ awareness on the importance and cost-effectiveness of six-month exclusive breastfeeding [25]. The project was widely recognized and led to cross sectoral support through a Memorandum of Understanding (MOU) signed by seven ministries and organizations in 2016 [26]. Currently, more than 1000 workplaces have established and implemented a breastfeeding corner. These programmes have shaped public attitudes that breastfeeding takes society-wide collective action, and is not the sole responsibility of mothers.

#### Breastfeeding protection

The MOPH adopted the BMS Code as a voluntary measure in 1984, then updated it to the second Thai Code in 1995 as part of BFHI guidelines in order to promote compliance to the Code among healthcare professionals. The third Thai Code through a Ministerial Notification in 2008 prohibited all marketing promotion of BMS products in public health facilities [23, 27]. The three non-enforceable voluntary Codes were replaced by legislation in national Law; the Control of Marketing Promotion of Infant and Young Child Food Act. B.E.2560 in 2017 [27].

The Labour Protection Act B.E. 2541 was enacted in 1998 to provide 90-day maternity leave for female employees [28]. Later on, paid maternity leave was extended to 98 days, in compliance with the minimum requirement of the Maternity Protection Convention number 183, by amendment of Labour Protection Act B.E. 2562 [29].

**Fig. 3:**
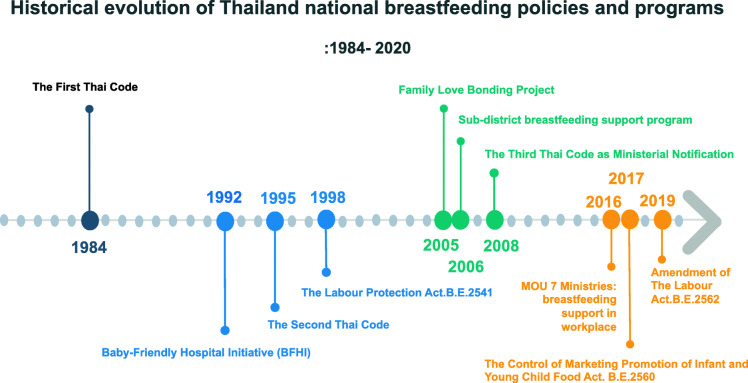
Historical evolution of Thailand national breastfeeding policies and programs, 1984–2020

Concerning the historical evolution of breastfeeding in Thailand, we found that the promotion and support of breastfeeding was implemented in a phased manner and gradually gained a solid foundation, while the implementation of breastfeeding protection took longer for policy adoption and implementation. The ineffective three voluntary BMS Codes were eventually replaced by a Law. Legislating the Code into a national law required strong social mobilization and political commitment to fight the lobbying power of BMS industries and their proxies [27]. Figure 3 describes the historical evolution of national breastfeeding policies and programmes.

### Breastfeeding practices and trends: potentially off-track to achieve global target

Despite efforts to promote, support and protect, the exclusive breastfeeding rate in Thailand is constantly low and has never achieved either national or global targets. During 1969–1979, evidence showed a steady decline in the duration of breastfeeding among Thai women, in particular breastfeeding was least commonly practiced by women in Bangkok, and most extensively practiced among women in Northeast region. Breastfeeding was noticeably lower among urban, higher educated, and wealthier women than those who live in rural areas, are less educated and poorer [30]. The national survey in 1981 confirmed the decline of breastfeeding duration with substantial differences among rural-urban women, their educational levels, and location in geographical regions. The survey also showed that Thai mothers introduced supplementary food such as rice mixed with fruit and eggs as part of a child’s feeding within the first few months of life [31].

The national survey conducted later in 1995, 1998, 2000, and 2005 found that the rate of four-month exclusive breastfeeding was very low as the majority of mothers fed their babies water in addition to breast milk [23]. In 2002, Thailand began to monitor the rate of exclusive breastfeeding by using a 24-h recall period, changing from the previous pattern that asked mother directly about the duration of exclusive breastfeeding. A series of national surveys, Multiple Indicator Cluster Survey (MICS) was conducted in 2005–2006 [32], 2012 [33], 2015–2016 [6], and 2019 [34].

**Fig. 4:**
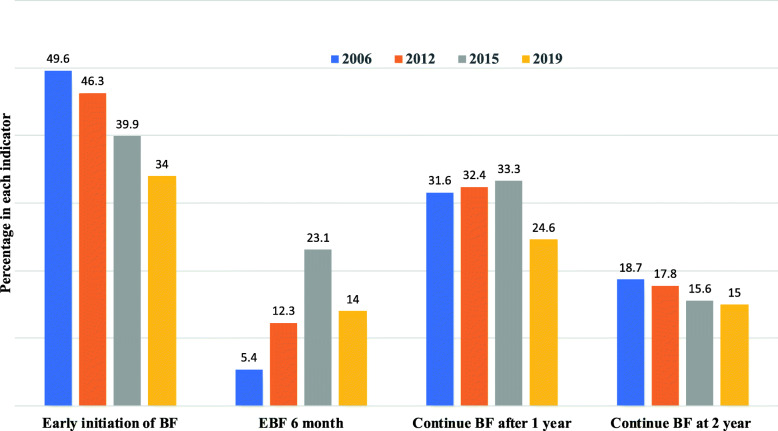
Thailand breastfeeding trends between 2006 and 2019. Source: Thailand MICS 2006, 2012, 2015 and 2019 [6, 32–34]

The result from MICS revealed gradual improvement of the six-month exclusive breastfeeding rate for the last decade, Table 1. However, there was a sharp decline in the latest MICS in 2019, in combination with a gradual decrease of the breastfeeding initiation rate within 1 h after birth and continued breastfeeding beyond one and 2 years, see Fig. 4. The 2019 MICS results clearly showed that Thailand is unlikely to achieve the 2025 target, unless significant efforts are in place. Additionally, substantial differences in breastfeeding practices among women according to urban-rural, geographical regions, educational levels, and income levels still exist. Nonetheless, a reverse relationship was identified in more recent surveys showing that women with the highest educational and income status tend to breastfeed more than other groups [6].

**Table 1:** Percentages of exclusive breastfeeding rate of infants during the 6 months against national and global targets, 1993–2019

Year	1993	1995	2000	2002	2005	***2006***	***2012***	***2016***	***2019***
**EBF 4 months**	1.3	3.6	2.92	13.8	20.7				
**EBF 6 months**					14.5	*5.4*	*12.3*	*23.1*	*14.0*
**National Target**	EBF 4–6 months; 15 by 2001				EBF 6 months; 30 by 2006 [35]		50		
**Global Target**							EBF 6 months; 50 by 2025 [16] 70 by 2030		

Source: data [6, 23, 32–34]

- 1993 from Family Health Division

- 1995 from Nutrition Division, Department of Health, MOPH

- 2000, 2002, and 2005 from Department of Health, MOPH

- *2006, 2012, 2016, 2019 from MICS*

The low level of breastfeeding practices among Thai women leads to negative health outcomes which contributes to poor national and socioeconomic development. An estimation shows that inadequate breastfeeding in Thailand resulted in 600 child deaths from diarrhoea and pneumonia; 1180 maternal deaths from breast and ovarian cancers; approximately 600 million USD household spending on infant formula milk and related products, and; almost 6 million USD (almost 0.001% of Gross National Income) of national health expenditure due to treatment of child illnesses [36].

### Gap analysis

**Fig. 5:**
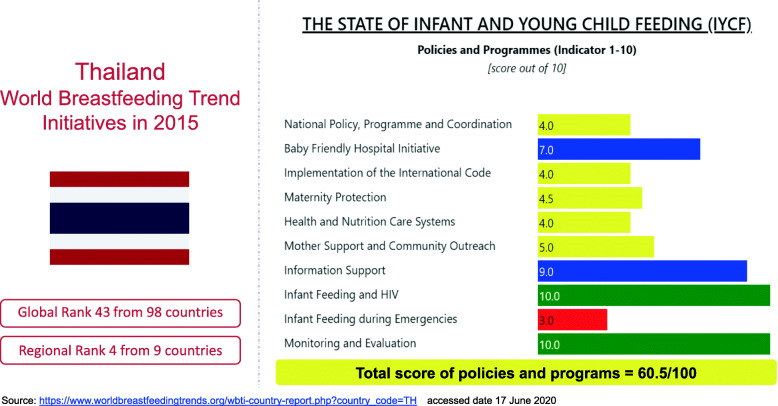
Policy and programmes on infant and young child feeding, Thailand 2015. Source: WBTI assessment report: Thailand 2015 [38]

Despite programme implementation that promotes, supports and protects breastfeeding, Thailand’s performance remains poor. In 2015, out of total 98 countries, Thailand was ranked 43rd (score 60.5) by the World Breastfeeding Trend Initiative (WBTi) (the first rank is the top of the world) [37]. The WBTi-country assessment revealed that despite Thailand making progress in many aspects of its breastfeeding policy compared with the 2010 assessment, policy gaps existed. In particular, an absence of national policy or strategy on IYCF and national coordination, ineffective enforcement of existing regulations on IYCF and health and nutrition care systems including infant feeding guideline during emergencies, and inadequate maternity leave for working mothers [38], see Fig. 5.

This review identifies four gaps, which are mostly related to inadequate investment, ineffectiveness of intervention, and weak law enforcement in the context of aggressive market promotion by BMS industries.

Firstly, the lack of comprehensive policy and strategies and programme development and evaluation lead to incoherent policy implementation across government sectors. Inadequate investment on breastfeeding, − 0.02 USD per child against the total requirement of 5 USD - is one of the major challenges [3, 39]. Even with additional UNICEF funding for breastfeeding and the government’s monthly child allowance to low-income households, 600 Thai Baht is far too small compared with the 5 USD needed. Advocating breastfeeding requires adequate resources to support multi-sectoral actions for health and media campaigns. While the government spends too little, BMS companies spend much more on marketing their products and this significantly exceeds government spending [40].

Secondly, an absence of legal measures resulted in the unsuccessful control of BMS advertising which shaped mothers’ attitudes in favour of breastmilk substitutes [41]. Evidence demonstrates the ineffective enforcement of the three voluntary Thai BMS Codes in 1984, 1995 and 2008, there were no legal sanctions to the violators [42, 43]. Even though Thailand has successfully enacted the Control of Marketing Promotion of Infant and Young Child Food Act. B.E.2560 in 2017 [44], effective enforcement remains a major challenge. To ensure an effective enforcement of the Act, the national committee, as mandated by the Act, has adopted a three-year plan for regular monitoring and law enforcement during 2020–2022. The outcome has yet to be monitored by all stakeholders including civil society organizations [45].

Thirdly, after several years of the BFHI and FLBP implementation, they were integrated into routine practices by all maternity wards in public hospitals, but lost momentum as there were neither incentives nor an accreditation system. The compliance to BFHI practices dropped even among the BFHI accredited hospitals. Other priorities such as teenage pregnancy and child development, compete for attention and resources. The decline of the BFHI implementation was reflected by the country assessment that only 61% of births took place in Baby-Friendly Hospitals [46]. The downward trend of early initiation of breastfeeding immediately after birth was a good indicator showing inadequate support from hospital. A high number, 99%, of pregnant women gave birth in hospital in 2016 [6]. Additionally, a sub-district breastfeeding support programme was toned down by other campaigns such as the ‘1000 days sub-district project’ as well as lack of funding or human resources to support community-based breastfeeding intervention. As a result, many mothers who encountered breastfeeding difficulties received no support and were at risk of exclusive breastfeeding failure [35, 47].

Lastly, Thailand’s female participation rate in the labour force was high; 59% compared with an average 54% in upper-middle income countries [48]. Evidence showed that mothers returning to work faced many difficulties especially finding an appropriate time and place for expressing breast milk during working hours, when some supervisors and co-workers discouraged them to continue breastfeeding practice [35]. Mothers working in informal private sectors received no maternity leave and those in lower position were most vulnerable as they could not manage their time as needed. With these contextual environments, the 98 days maternity leave with full pay (combined employers and Social Security Scheme) is inadequate to support six-month exclusive breastfeeding. Furthermore, workplace environments have yet to be conducive for breastfeeding. However, research found that extending maternity leave to 6 months, with the full pay of 98 days may lead to mothers’ low compliance for economic reasons [49] and the fear of losing their job, especially in the context of an economic downturn from the COVID-19 pandemic. Therefore, promoting breastfeeding-supportive environments in workplace could be a feasible and practical option for maintaining breastfeeding practice as well as promoting job security for all women.

### The ways forward to achieve 2030 targets

Given the health and socioeconomic benefits from breastfeeding and key implementation gaps, it is essential that the Thai Government spares no effort on breastfeeding protection, promotion and support. Although achieving the 2030 target for Thailand is ambitious, to ensure the realization of the child’s right to breastfeed, a few actions are recommended.

Firstly, establish a national comprehensive plan at the level of MOPH, with well-resourced support, on breastfeeding protection, promotion, and support; and strengthen the monitoring and evaluation system with feedback for policy adjustment.

Secondly, strengthen monitoring and public reporting of BMS companies which have low or non- compliance, and boost the regulatory capacity of the Department of Health, MOPH, which is responsible for implementation of the Act in order for it to take tougher measures. The adoption of stricter regulatory frameworks coupled with independent, quantitative monitoring and compliance enforcement are needed to counter the negative impact of formula marketing [50]. The aggressive social marketing and online advertisement of infant formula requires special attention.

Thirdly, ensure that all mothers are supported to have uninterrupted skin-to-skin contact and initiate breastfeeding immediately after birth through the strengthening of BFHI and ensuring sustainability over time. This could begin with the assessment of the current BFHI compliances, and some practices may need revision and adaptation to be more feasible and comprehensive. The MOPH works with the health professional bodies such as the Royal Thai College of Obstetricians and Gynaecologists, the Royal College of Pediatricians of Thailand, and Thailand Nursing and Midwifery Council, to adequately provide and standardize breastfeeding management in the pre-service and in-service training curriculum.

Finally, civil society and women’s organizations should advocate for more supportive workplace environments, and at least ensure that all workplaces provide enough break time for mothers to express and store their breast milk. In parallel, academia could provide evidence on the benefits and feasibility of six-month maternity leave when the political window of opportunities opens.

## Conclusions

Breastfeeding has been championed as a global public health agenda for decades. Successful breastfeeding requires concerted efforts at local, national and global levels; therefore, a series of global recommendations to promote, support and protect breastfeeding were developed. Although the Royal Thai Government has demonstrated its commitment through the implementation of breastfeeding policies and programmes in line with global recommendations, progress has been slow. Thailand has yet to translate breastfeeding policies and programmes into effective implementation through increased investments of funding, systems, human resources and regulatory capacity. Further, a comprehensive strategy for achieving the breastfeeding target has not been established which would help to frame multi-sectoral actions.

To achieve improved breastfeeding indicators, key recommendations are provided: increase investment on breastfeeding through family, health and welfare systems, and workplace programmes; increase effective enforcement of the Control of Marketing Promotion of Infant and Young Child Food Act. B.E. 2560; revitalize the BFHI implementation in public hospitals and extend to private hospitals; and promote breastfeeding support programmes in workplaces and communities.

## Data Availability

Not applicable as this is a review.

## Abbreviations

EBF: Exclusive Breastfeeding
BFHI: Baby-Friendly Hospital Initiative
BMS Code: The International Code of Marketing of Breast-Milk Substitutes
CRC: The Convention on the Right of the Child
FLBP: Family Love Bonding Project
MICS: Multiple Indicator Cluster Survey
MOPH: Ministry of Public Health, Thailand
WBTI: World Breastfeeding Trend Initiative
WHA: World Health Assembly
